# Near-infrared auto-fluorescence spectroscopy combining with Fisher’s linear discriminant analysis improves intraoperative real-time identification of normal parathyroid in thyroidectomy

**DOI:** 10.1186/s12893-019-0670-x

**Published:** 2020-01-06

**Authors:** Junsong Liu, Xiaoxia Wang, Rui Wang, Chongwen Xu, Ruimin Zhao, Honghui Li, Shaoqiang Zhang, Xiaobao Yao

**Affiliations:** 1 Department of Otorhinolaryngology-Head and Neck Surgery, 277 West Yanta Road, Xi’an, Shaanxi 710061 People’s Republic of China; 2 Department of Otorhinolaryngology, Air Force 986 Hospital of Chinese People’s Liberation Army, Xi’an, Shaanxi 710054 People’s Republic of China; 3 Department of Anesthesiology, The First Affiliated Hospital of Xi’an Jiaotong University, 277 West Yanta Road, Xi’an, Shaanxi 710061 People’s Republic of China

**Keywords:** Parathyroid, Near-infrared, Auto-fluorescence, Thyroidectomy

## Abstract

**Background:**

To evaluate the efficacy of a sensitive, real-time tool for identification and protection for parathyroid glands during thyroidectomy.

**Methods:**

Near-infrared (NIR) auto-fluorescence was measured intraoperatively from 20 patients undergoing thyroidectomy. Spectra were measured from suspicious parathyroid glands and surrounding neck tissues during the operation with a NIR fluorescence system. Fast frozen sections were performed on the suspicious parathyroid glands. Accuracy was evaluated by comparison with histology and NIR identification. Data were attracted for Fisher’s linear discriminant analysis.

**Results:**

The auto-fluorescence intensity of parathyroid was significantly higher than that of thyroid, fat and lymph node. The peak intensity of auto-fluorescence from parathyroid was 5.55 times of that from thyroid at the corresponding wave number. Of the 20 patients, the parathyroid was accurately detected and identified in 19 patients by NIR system, compared with their histologic results. One suspicious parathyroid did not exhibit typical spectra, and was proved to be fat tissue by histology. The NIR auto-fluorescence method had a 100% sensitivity of parathyroid glands identification and a high accuracy of 95%. The positive predictive value was 95%. The parathyroid gland have specific auto-fluorescence spectrum and can be separated from the other three samples through the Fisher’s linear discriminant analysis.

**Conclusions:**

NIR auto-fluorescence spectroscopy can accurately identify normal parathyroid gland during thyroidectomy. The Fisher’s linear discriminant analysis demonstrated the specificity of the NIR auto-fluorescence of parathyroid tissue and its efficacy in parathyroid discrimination.

## Background

With the increasing incidence of thyroid nodule, of which thyroid carcinoma accounts for 7–15%, there is a big rise in the amount of patients undergoing thyroidectomy [1]. One of the main concerns during thyroidectomy is the in situ preserving and protection of parathyroid glands which account for the calcium homeostasis. Data showed that the incidence of inadvertent removal of parathyroid glands during thyroidectomy ranges 8–19%, depending on the surgeon’s experience and the type of surgical procedures [2]. Accidental removal or injury of the parathyroid glands during thyroidectomy may lead to temporary or permanent postoperative hypoparathyroidism and hypocalcemia, and subsequently affect the patients’ quality of life [3–5]. Nevertheless, there was no definite evidence to confirm that parathyroid implantation could decrease the probability of permanent hypoparathyroidism when parathyroid glands were accidental removal or could not be preserved in situ [6, 7].

It is the characteristics of parathyroid itself that makes parathyroid difficult to be distinguished from other cervical tissues [8]. The size of a normal parathyroid gland is too small, and its appearance is similar to the surrounding anatomic structures such as lymph nodes, fat and thyroid tissue. Moreover, the parathyroid glands vary in location and are often covered by overlying layers of fat. These facts make it difficult for surgeons, especially those inexperienced ones, to visually discriminate normal parathyroid from other cervical tissues.

Currently, surgeons mainly rely on visual inspection to identify parathyroid during thyroidectomy, which is subjective and inconclusive. Ultrasound, computed tomography (CT) and magnetic resonance imaging (MRI) are just used for preoperative diagnosis of diseased parathyroid glands. Biopsy of the gland requires partial excision of a gland and thus leads to damage to its blood supply and destruction of gland function [9]. Fine-needle aspiration with intact parathyroid hormone (PTH) is time-consuming and suffers from its low sensitivity [10]. Utility of indocyanine green (ICG) fluorescence imaging for intraoperative localization needs exogenous contrast agent [11, 12]. Serum parathyroid hormone (PTH) measurement is mainly for postoperative evaluation and thus not timely enough. So, it is urgent for us to look for a rapid, real-time, precise and noninvasive method for intraoperative identification and protection of parathyroid glands.

In recent years, Melanie A. McWade and colleagues reported their use of Near-infrared (NIR) auto-fluorescence for the detection of parathyroid glands during thyroidectomies and parathyroidectomies, and achieved promising results [13–15]. Data showed that auto-fluorescence from parathyroid, exited by 785 nm near-infrared laser, was stronger than those from other cervical tissues like thyroid, fat, muscle and trachea. And the relative intensity of peak parathyroid fluorescence at 820-830 nm was about 1.2 to more than ten times that from thyroid. The specific near-infrared spectrum of parathyroid and visualization of auto-fluorescence can be transformed into an efficient tool for detection of parathyroid glands. De Leeuw F [16] and Kim SW [17] also got similar, encouraging results and demonstrated that NIR imaging technique based on parathyroid auto-fluorescence could be a simple, fast, safe, and non-invasive method for helping surgeons precisely locate and preserve parathyroid glands during thyroidectomies. Recently, a multicenter analysis including 210 patients undergoing thyroid or parathyroid surgery showed that the NIR auto-fluorescence imaging helped detect 37–67% parathyroid glands which were not identified by the naked eyes due to coverage by soft tissue [18]. Another study showed that the use of NIR imaging technique increased the number of identified parathyroid glands and therefore decreased postoperative hypocalcemia rate, from 20.9 to 5.2%, demonstrating the value of NIR imaging method in thyroidectomies [19].

In the present study, we detected the healthy parathyroid glands using NIR auto-fluorescence during thyroidectomies in the Chinese population and provided further evidence. We, for the first time, performed the Fisher’s linear discriminant analysis for statistical illustration for the use of NIR auto-fluorescence in parathyroid identification. And data analysis confirmed the specificity of the NIR auto-fluorescence of parathyroid tissue and demonstrated the values of this non-invasive procedure in clinical use.

## Materials and methods

### Patients and methods

Patients with primary thyroid tumors undergoing partial or total thyroidectomy at the Department of Otorhinolaryngology-Head and Neck Surgery, The First Affiliated Hospital of Xi’an Jiaotong University were included in the study. Approval was obtained from the Institutional Review Board. Informed written consent was obtained from each enrolled patient regardless of gender or age. Patient eligibility was evaluated before operation.

### Fluorescence measurements

For each patient, the surgeon operated according to the standard procedures of thyroidectomy with or without lymph node dissection, and tried to find out and identify the parathyroid glands by experience. Once the surgeon was not certain of the parathyroid glands during the surgeries, fast frozen sections were performed on the suspicious parathyroid glands. Before incisional biopsy, we measured the auto-fluorescence spectra of the suspicious parathyroid glands and other certain cervical tissues using an NIR fluorescence spectroscopy system. The NIR judgement was compared with the histologic results. When the parathyroid was confirmed, we just kept the residual glands where they were if the parathyroid glands remained in situ. If the glands were removed unexpectedly, the residual gland tissues were autotransplanted into the sternocleidomastoid muscle. During the waiting period for biopsy results, the residual gland tissues were kept in cold normal saline.

*i*-Raman® Pro near-infrared (NIR) system (Bedtech, inc) was used for auto-fluorescence detection. Tissue was excited with 80 mW of light and fluorescence measurement takes 1 s, keeping the operating lights turned off. Each tissue sample was measured for three times to reduce the measurement errors. Subcutaneous fat and thyroid tissue were easily confirmed visually and thus were measured firstly as baseline. Then, suspicious parathyroid glands and lymph nodes were measured. Data were collected for analysis.

### Data processing and statistical analysis

Statistical analysis was performed with a *Student’s t* test using SPSS 16.0(SPSS Inc., Chicago, IL, United States) to detect significant differences in the intensity of fluorescence from parathyroid and other cervical tissues. *P <* 0.05 was considered statistically significant. Data of fluorescence were attracted for the Fisher’s linear discriminant analysis and the process was performed by machine learning.

## Results

### Validation of parathyroid auto-fluorescence with pathology

Twenty patients, aging from 36 to 69 years, were included in the study. Of these patients, 12 (60%) were diagnosed with benign thyroid nodules and underwent partial thyroidectomy, and 8 (40%) were diagnosed with papillary thyroid cancers and underwent partial or total thyroidectomy plus central neck lymph node dissection.

**Fig. 1:**
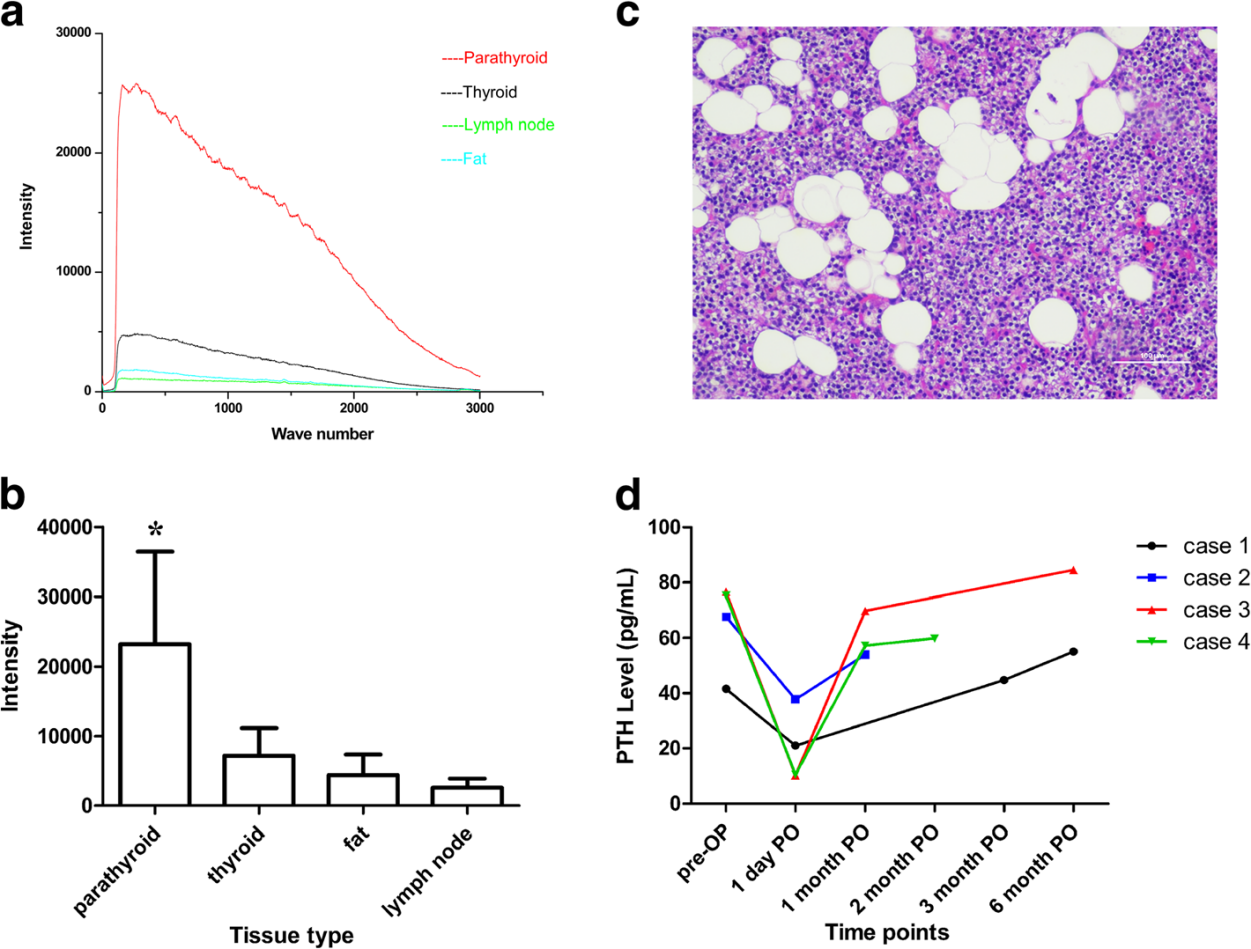
**a** Typical normalized NIR spectra of parathyroid and other cervical tissue (thyroid, fat and lymph node). The peak wave number ranges 267-281 cm^− 1^. The auto-fluorescence intensity of parathyroid is stronger than that of other tissues in the neck. **b** Comparison of peak auto-fluorescence intensity from the above cervical tissues. * The peak intensity of auto-fluorescence from parathyroid gland is significantly stronger than those from thyroid, fat and lymph node. **c** Representative histologic section of parathyroid gland tissue. (plotting scale represents 100 μM). **d** The recovery courses of thyroid function of four thyroid carcinoma patients undergoing total thyroidectomy with central neck dissection. Pre-OP: pre-operation; PO: post-operation; PTH: parathyroid hormone. Normal reference range of PTH: 15~65 pg/mL

Measurements were performed in the 20 patients undergoing thyroidectomy. The NIR auto-fluorescence detection process took no more than 1 min each time. The auto-fluorescence intensity of parathyroid was significantly stronger than that of thyroid, fat and lymph node. The peak intensity of auto-fluorescence from parathyroid was 5.55 times that of thyroid at the corresponding wave number (267-281 cm^− 1^) (Fig. 1a, b). Of the 20 patients, the parathyroid was accurately detected and identified in 19 patients by NIR system, compared with their histologic results. One suspicious parathyroid did not exhibit typical spectra, and was proved to be fat tissue by histology. Thus, the NIR auto-fluorescence method had a 100% sensitivity of parathyroid glands identification and a high accuracy of 95%. The positive predictive value was 95%. Two suspicious lymph nodes were proved to be thyroid tissue and one suspicious lymph nodes was proved to be fat tissue by histology. The results indicate that the NIR auto-fluorescence can detect the normal parathyroid with high accuracy in real time. The representative histologic section of parathyroid gland tissue was shown as Fig. 1c.

Of the 20 included patients, four patients underwent total thyroidectomy with central neck lymph node dissection. They just experienced transient hypoparathyroidism. Three patients recovered within 1 month post-operation, and one recovered at 3-month post-operation (Fig. 1d).

### The Fisher’s linear discriminant analysis demonstrated the specificity of the NIR auto-fluorescence of parathyroid tissue and its efficacy in parathyroid discrimination

Data of auto-fluorescence of the four type tissues were attracted from 20 patients and 80 samples. The samples those were not correctly diagnosed by NIR system and those without typical NIR spectra were excluded, and 68 samples were finally used for analysis. Through preliminary analysis, we found that the intensity differences between the four tissues diminished gradually with the increase of wave number. So, 402 dimensions of each data were used for final analysis. We used Fisher’s linear discriminant analysis to discriminate parathyroid and the other three type tissues.

**Fig. 2:**
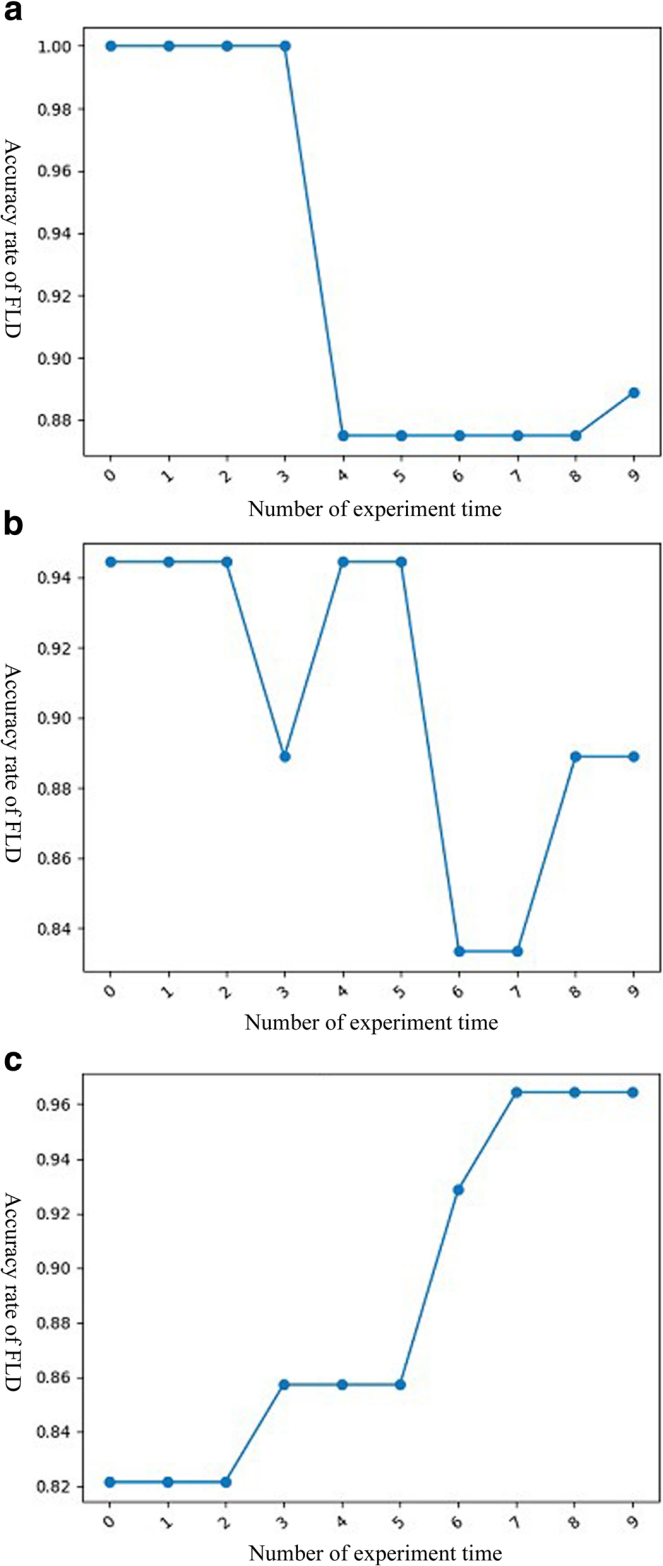
The Fisher’s linear discriminant analysis results presented as accuracy with different times. **a** When taking 60 data samples as training samples and 8 data as test samples, the average accuracy rate was 92.64%. **b** When taking 50 data samples as training samples and 18 data as test samples, the average accuracy rate was 90.05%. **c** When taking 40 data samples as training samples and 28 data as test samples, the average accuracy rate was 88.57%

Cross-validation method was used, a portion of data used as training samples and the rest of data used as test samples. Ten time experiments were performed to get the average classification accuracy. When taking 60 data samples as training samples and 8 data as test samples, the average accuracy rate was 92.64% (Fig. 2a). When taking 50 data samples as training samples and 18 data as test samples, the average accuracy rate was 90.05% (Fig. 2b). When taking 40 data samples as training samples and 28 data as test samples, the average accuracy rate was 88.57% (Fig. 2c). Our result indicated that the parathyroid glands have specific auto-fluorescence spectra and can be separated from the other three tissues through the Fisher’s linear discriminant analysis. More amounts of data will produce better accuracy.

## Discussion

Parathyroid gland accounts for calcium regulation. Due to its special appearance and the anatomical adjacency to thyroid, the identification and protection of parathyroid glands is a key process in the thyroidectomy procedure. Unintentional removal or injury of the parathyroid glands during thyroidectomy may lead to temporary or permanent hypoparathyroidism and hypocalcemia, and lead to numbness in the hands and feet, even tetany. The incidence of permanent hypocalcemia after total thyroidectomy is about 0–3.8% [20]. This clinical problem would eventually lead to fatal convulsions, cardiac arrhythmia, heart failure and chronic renal failure [3, 21]. For patients with thyroid carcinoma, if the lymph node were mistaken as parathyroid gland, it would give rise to the risk of cancer residual and increase recurrence rate. So, a noninvasive and timely procedure is indispensable during thyroidectomy so as to identify the parathyroid glands accurately.

In the present study, we present that NIR fluorescence spectroscopy can accurately identify normal parathyroid glands without additional injury to its function. Each sample was validated by histologic result. Moreover, we designed a Fisher’s linear discriminant model which provided reliable verification for the data of NIR auto-fluorescence. Fisher’s linear discriminant analysis is a useful model for the distinction of objects that belong to different classes. It extracts descriptive characteristics, and is used to reduce the dimensionality of a space, to generate a discriminant space with a lower dimension, keeping useful information to discriminate among classes [22–24]. In the present study, we used Fisher’s linear discriminant is to discriminate auto-fluorescence spectra from parathyroid glands and other cervical tissues and achieved an accuracy about 90%. To our knowledge, this is the first report of Fisher’s linear discriminant model used for predicting parathyroid glands by detection data and reveals high accuracy. The results indicate that NIR fluorescence spectroscopy can provide real-time, reliable and repeatable help for surgeon during thyroidectomies. Additionally, the combination of optical metrology model with NIR technique provides the potential for transforming it to an artificial intelligent detection instrument, making this technique easier and faster for practical use.

NIR fluorescence spectroscopy is a novel and promising method for intraoperative detection of parathyroid gland during thyroidectomy. Compared with other parathyroid identifying methods, especially biopsy or fine-needle aspiration with intact PTH, the greatest advantages of the tool using intrinsic NIR auto-fluorescence are noninvasive and time-saving. It does no extra injury to the parathyroid glands and thus provides protection for the parathyroid function. The whole detection procedure just takes a few minutes without affecting the duration of surgery.

The tool uses intrinsic NIR auto-fluorescence to distinguish different tissues. However, the underlying molecular basis is still not clear. Primary hypothesis considers tissue specific fluorophore, the extracellular Calcium-sensing Receptor (CaSR). The CaSR is a G-protein-coupled receptor mainly expressed in tissues participating Ca^2+^ regulation, including parathyroid chief cells, thyroidal C-cells, kidney, bone and intestinal cells at different levels but not present in other neck tissues [25, 26]. The tissue specific distribution and expression level of CaSR fluorophore make it possible that parathyroid emits higher intensity of NIR auto-fluorescence when exited by certain length of NIR laser. McWade and colleagues detected the same peak fluorescence signal in human kidney and colon when exited as parathyroid and thyroid [15]. Furthermore, the intensity of the fluorescence was consistent with the CaSR expression level. This provides primary evidence for the candidate role of CaSR in parathyroid NIR auto-fluorescence. Further cellular and spectroscopic studies will probably provide interpretation.

A potential restriction of the tool is that the detection depth is limited, because of the limitation of near infrared light penetration. When the parathyroid gland is covered by overlying layer of fat tissue, it may influence the accuracy of detection [27]. Another defect is the current NIR technique cannot estimate blood perfusion of parathyroid glands [18, 28]. Furthermore, the detection procedure requires turning off the operation lights [15, 29]. Further improving in the technology is still needed. The study of the underlying molecules and chemical construction, together with the use of artificial intelligent model, will provide the possibility.

In previous studies, Constantine Paras and colleagues have detected the near-infrared autofluorescence of pathologic parathyroid glands. When stimulated by 785-nm excitation, parathyroid fluorescence was several times stronger than that of the thyroid tissues with peak fluorescence occurring at 820 to 830 nm regardless of disease state. The results indicated that the near-infrared autofluorescence from pathologic parathyroid glands is similar to that from normal parathyroid glands [13, 14]. In the other hand, in patients with parathyroid diseases, such as parathyroid hyperplasia and parathyroid adenoma, the parathyroid glands usually become larger in size, round in shape, and increase in hardness. Thus, it is usually very easy to find out and identify the pathologic parathyroid glands without the need for extra tools in endocrine surgeries. The difficulty in clinical practice is the identification of normal parathyroid glands because of its small size and similarity in appearance to the surrounding tissues [8]. Based on the facts above, we focused on normal parathyroid glands identification and detection, and did not included patients with parathyroid diseases.

Overall, our study presents a practical technique tool and a predicting optical metrology model which would provide real-time help in detecting and protecting parathyroid glands in endocrine surgeries. We just did validation in twenty patients. The increase of sample amount will improve the efficacy of both the detection method and the metrology model. We believe that a portable and intelligent NIR instrument will decrease postoperative hypoparathyroidism and hypocalcemia, and improve patient outcome.

## Conclusions

NIR auto-fluorescence spectroscopy is a novel practical tool that can accurately identify normal parathyroid glands during thyroidectomy. The Fisher’s linear discriminant analysis demonstrated the specificity of the NIR auto-fluorescence of parathyroid tissue and its efficacy in parathyroid discrimination. Near-infrared auto-fluorescence spectroscopy combining with Fisher’s linear discriminant analysis improves intraoperative real-time identification of normal parathyroid glands in thyroidectomy.

## Data Availability

The datasets used and analyzed during the current study are available from the corresponding author on reasonable request.

## Abbreviations

CaSR: Calcium-sensing Receptor
NIR: Near-infrared
PTH: Parathyroid hormone
