# Correction to: Near-infrared auto-fluorescence spectroscopy combining with Fisher’s linear discriminant analysis improves intraoperative real-time identification of normal parathyroid in thyroidectomy

**DOI:** 10.1186/s12893-021-01071-z

**Published:** 2021-02-14

**Authors:** Junsong Liu, Xiaoxia Wang, Rui Wang, Chongwen Xu, Ruimin Zhao, Honghui Li, Shaoqiang Zhang, Xiaobao Yao

**Affiliations:** 1 Department of Otorhinolaryngology-Head and Neck Surgery, 277 West Yanta Road, Xi’an, 710061 Shaanxi People’s Republic of China; 2 Department of Otorhinolaryngology, Air Force 986 Hospital of Chinese People’s Liberation Army, Xi’an, 710054 Shaanxi People’s Republic of China; 3 Department of Anesthesiology, The First Affiliated Hospital of Xi’an Jiaotong University, 277 West Yanta Road, Xi’an, 710061 Shaanxi People’s Republic of China

## Correction to: BMC Surgery (2020) 20:4 https://doi.org/10.1186/s12893-019-0670-x

Following publication of the original article [1], we were notified of a few errors in partial data calculating and a spelling mistake in the footnote of Fig. 1d. These corrections do not change the main results and do not affect the conclusion in this article.

- In the study [1], a total of 20 specimens were tested by near-infrared (NIR) auto-fluorescence system and the results were compared with the histology results. Among the 20 specimens, 19 parathyroid specimens were accurately detected and identified by NIR system. One suspicious parathyroid did not exhibit typical spectra, and was proved to be fat tissue by histology. So, all the 19 parathyroid glands were identified and the fat tissue (one specimen) was not regarded as parathyroid gland by the near infrared system. So, the sensitivity, the accuracy and the positive predictive value of the near infrared system should be 100% (19/19), 100% (20/20) and 100% (19/19), respectively. Our results indicate that the NIR auto-fluorescence spectroscopy can identify normal parathyroid gland with high accuracy and sensitivity. The correction does not change the main results and does not affect the conclusion in the article. On the contrary, the data indicate a better efficacy of NIR auto-fluorescence spectroscopy in identification of normal parathyroid gland.
- In the footnote of Fig. 1d, thyroid was miswrote as parathyroid. The corrected footnote of Fig. 1d should be “The recovery courses of parathyroid function of four thyroid carcinoma patients undergoing total thyroidectomy with central neck dissection”.
